# Evaluating the Effects of Social Capital, Self-Stigma, and Social Identity in Predicting Behavioral Intentions of Agricultural Producers to Seek Mental Health Assistance

**DOI:** 10.3390/ijerph191912110

**Published:** 2022-09-24

**Authors:** Carrie N. Baker, Robert Strong, Carly McCord, Tobin Redwine

**Affiliations:** 1 Department of Agricultural Leadership, Education and Communications, Texas A&M University, 600 John Kimbrough Blvd, College Station, TX 77843, USA; 2 Department of Psychiatry and Behavioral Sciences, Texas A&M University, 2900 E. 29th Street, Bryan, TX 77802, USA; 3 Department of Educational Psychology, Texas A&M University, Health Professions Education Building, 8447 Riverside Pkwy, Bryan, TX 77807, USA

**Keywords:** occupational health, stress, mental health education, mental health literacy, help-seeking, agricultural extension, rural health

## Abstract

Mental illness significantly impacts agricultural producers, whose occupation puts them at increased risk for compromised mental health and related disorders. Help-seeking intention, which can be mediated by variables such as social identity, social capital, and self-stigma, can lead to improved mental health outcomes. This cross-sectional study aimed to describe the intention of agricultural producers to seek mental health assistance and determine whether these three variables are associated with help-seeking intention. Researchers administered a cross-sectional survey of agricultural producers from two regions in 32 Texas counties. Researchers surveyed a sample of Texas agricultural producers (*n =* 429) to understand their social identity, social capital, and degree of self-stigma, and their intent to seek help for personal or emotional problems and for suicide ideation. Researchers identified a relationship between social identity and social capital, which indicated that social identity is moderately associated with greater levels of social capital. The multiple linear regression analyses confirmed that social capital and self-stigma are significant predictors of producers’ help-seeking intention for both help-seeking types. These results signify the importance of efforts to increase social capital, increase mental health literacy and tailor training to address self-stigma and enhance positive help-seeking behavior among agricultural producers.

## 1. Introduction

A study published in 2020 by the Centers for Disease Control and Prevention (CDC) found that suicide rates among working populations are increasing [1]. Most notably, Peterson et al. found the Agriculture, Forestry, Fishing and Hunting industry was one of five major industry groups where suicide rates were significantly higher when compared to those in the total study population [2]. As a result, recent studies have examined rates of self-harm and suicide within the agricultural industry—many of which note that laborers, specifically farmers are at greater risk of suicide [1,2]. Others seek to investigate the farmer experience, in an attempt to better understand this phenomenon. Recent studies of Australian farmers identified significant factors influencing farmer health and wellbeing, citing rural living, remoteness, and financial stress as significant factors influencing farmers’ mental health [3]. Similar studies, conducted in Canada, France, and Australia investigated cultural factors associated with agriculture and the impacts of the farming lifestyle on the mental health of farmers, recognizing that many create barriers to seeking help or accessing mental healthcare [3,4,5,6,7]. However, few studies have attempted to characterize help-seeking or mental health decision-making behaviors of rural, agrarian populations, and even fewer have studied farmers in the United States. In leading states such as Texas, where agriculture is a major industry and agricultural producers represent a growing workforce, research on the mental health and well-being of agricultural producers is sparse [7,8]. A more sustainable and resilient agriculture industry to improve communities’ food security is plausible when the mental health of farmers is good [9].

Because farming is often seen as more than a job to agricultural producers, the farming lifestyle infiltrates many aspects of their life, including the people they surround themselves with. As social beings, individuals often operate within and ascribe to groups, which in turn, influence the way in which they conceptualize their identity. Klik et al. suggests that individuals begin to monitor and adjust their attitudes and behavior to align with group norms and beliefs [10]. This behavior, resulting from group assimilation, forms the foundation for social identity theory and its interaction with decision making, such as help-seeking [11]. Research by Polain et al. found that senior agricultural producers over the age of 58 opposed seeking help for their mental health because of cultural barriers relating to their social identity [12]. While social identity might be seen as a barrier to help-seeking where health promoting behavior is not encouraged, research also suggests that other dimensions of social influence on behavior, such as social capital found within identity groups, can counteract this negative influence. Developing target audiences’ trust is critical to diffusing interventions aimed at improving health [13].

Social capital is multidimensional and examines an individuals’ relationships, networks, and various systems of impact [14]. It can most easily be defined, as the various social connections in one’s life that could contribute to an individuals’ support system, including their degree of belongingness, proximity, and perceived reciprocity [14]. Magson et al. provides evidence suggesting that individuals with greater social capital have more positive mental health outcomes and have greater capacity to cope with stress, illness, and depression [14]. Further, recent research indicates that agricultural producers are most likely to seek help from those closest to them, (e.g., spouses and intimate partner, friend, relative) which emphasizes the importance of social capital and, more specifically, these individuals in a support network [8]. Research shows that Texas producers have high levels of social capital and rely closely on their familial contacts and those in their inner circle for support [8].

Studies, such as those conducted with European adults, students and adolescents in the rural South, suggest that higher measures of social capital counteract self-stigma (or negative attitudes and beliefs about mental illness that have been internalized) breaking down barriers for help-seeking [15,16,17]. Due to feelings of being less than or unworthy, individuals will often conceal their mental illness or refuse help due to fear or shame they self-associate with the label of mental illness [18]. While stigma research is abundant in the literature, the examination of self-stigma among agricultural producers, specifically within the context of help-seeking, is nearly nonexistent. Recent research by Baker et al. suggests that rural Texas producers are willing to seek professional mental health assistance, despite the existence of barriers such as stigma, accessibility, and availability, providing support for further investigation of producers’ help-seeking behavior [8].

Decisions by farmers to adopt new programs and innovations are multidimensional as farmers are sometimes cautious of change [19]. Evidence suggests that a major determining factor of behavior is an individual’s intent to engage in that behavior [20]. While previous literature indicates a relationship between these variables might exist and provides support for their potential effect on help-seeking, there is a gap in the literature where these variables collectively. Therefore, studies examining the intent of U.S. agricultural producers to engage in help-seeking behavior and those seeking to understand the effect of identity, social capital and self-stigma on help-seeking behaviors are necessary contributions to the current body of research.

The purpose of this study was to determine the relationship between social identity, social capital, and self-stigma and explore how these variables effect Texas agricultural producers’ help-seeking intentions for their mental health.

## 2. Materials and Methods

Researchers administered a cross-sectional survey of agricultural producers across two regions in 32 Texas counties. Of those counties included, 75% (*n* = 24) are classified as rural counties, according to the Texas Department of State Health Services [21]. Researchers surveyed Texas agricultural producers (*n* = 429) to investigate their social identity, social capital, and degree of self-stigma, and their intentions to seek help for personal or emotional problems and for suicide ideation.

### 2.1. Study Sample

This study utilized purposive sampling to target Texas producers, ages 18-89, using databases of Agriculture and Natural Resources (ANR) Extension agents in the West and East Texas A&M AgriLife Extension Regions. “Producers” or “Agricultural Producers” was operationally defined using the definition provided within 7 CFR 4284.902, the subpart of Title 7-Agriculture of the Code of Federal Regulations [22]. The survey was emailed to 5137 agricultural producers, but because 92 were undeliverable, the resulting population was 5045 individuals. Of those potential participants, researchers achieved a sample size of 429 participants, resulting in a 8.50% response rate. Researchers conducted a t-test of early to late respondents to control for bias due to nonresponse [23,24]. There was no statistically significant differences in their responses, indicating that in this instance, nonresponse did not translate to bias, providing support for the extrapolation of data to a broader audience [23].

Of the respondents who chose to report gender (*n* = 300), there were 218 males, 79 females and three who preferred not to identify. In Texas, where roughly 38% of agricultural producers are women, the female response fell slightly below the demographic makeup of the state’s agricultural producers [25]. The majority of respondents (44.7%) who indicated their age (*n* = 235) were 65 years of age or older, followed by those ages 45–64 (42.1%) and 25–44 years (13.2%). There were no participants younger than 25 years of age. The sample was slightly older than the average producer in Texas, where the average age is 59.2 and the majority are between the ages of 45 and 64 [26]. Of those participants who reported marital status (*n* = 299), 79.6% of participants were married, 8.0% were single, 7.0% were divorced and the remaining 5.4% were widowed. Participants were also asked questions pertaining to the number of years working as an agricultural producer and their occupational classification. Of the respondents, 77.2% had worked 11 years or more (*n* = 298) and the majority reported their occupational classification as part-time (*n =* 344). These characteristics also reflected census data, which show that the majority of producers work part time and have also worked on the farm for 11 years or more [26]. The final demographic variable investigated was the sector and commodity group of the sample (*n* = 429). The top livestock or livestock products reported by producers was cattle and calves (*n* = 223) and goats (*n* = 40). The majority of crops produced was hay or haylage (*n* = 153) and wheat (*n* = 43). This also closely represents farm makeup in Texas, where the majority of farms have inventory of cattle and calves and hay and wheat make up the second and third most farm acreage, followed only by cotton [27]. One can see that while purposive sampling was utilized, producer characteristics of this sample closely mirrored the demographic makeup of a larger population of Texas agriculture producers, as shown through state level data for Texas in the 2017 Census of Agriculture [25,26,27].

### 2.2. Measures

A cross-sectional survey consisting of four pre-existing scales assessing four variables as well as a section for demographic information was developed to measure social identity, social capital, self-stigma, help-seeking intention and producer characteristics. Pre-existing scales for all constructs were adapted for use in the instrument. These scales included the Collective Occupational Identity Construct (COIC) [28], Personal Social Capital Scale (PSCS) [29], Self-Stigma of Seeking Help Scale (SSOSH) [30], and the General Help-Seeking Questionnaire (GHSQ) [31]. For this study, farmers’ social identity, or the extent to which they identify as part of a group of agricultural producers was measured using the COIC. This scale helped gauge the relevance and salience of farmer identity on behavior. The PSCS was used to measure the scope of producers’ perceived support networks including the identity and closeness of their connections. Self-stigma, or the degree of internalized stigma (negative attitudes and beliefs) about one’s own potential experience seeking help for mental health-related needs, was measured using the SSOSH; and the GHSQ was used to measure the intent of producers to seek help and likely sources of support for emotional problems and suicide ideation. Demographic information collected included gender, age, marital status, years working as a producer, occupational classification (i.e., hobby, part-time, or full time) and sector or commodity involvement, which were sourced from the National Agricultural Statistics Service [26].

Face validity of the adaptations were reviewed by university faculty with expertise in communication, agricultural science, and psychology with attention to survey research methods and Dillman et al.’s instrument design best practices (e.g., clear instructions before scales, navigational cues, white space, consistent formatting of items and responses, etc.) to make the survey easy for respondents to follow and answer [32]. Upon completion of data analysis, reliability was confirmed for this sample using Cronbach’s alpha [33]. Given their alpha coefficients, the PSCS (α = 0.94), GHSQ (α = 0.89), SSOSH scale (α = 0.86), and COIC (α = 0.82) were deemed reliable measures.

### 2.3. Procedures and Data Collection

Researchers utilized Qualtrics to develop the survey and administered it via email to potential participants using Dillman et al.’s tailored design method to increase response rate [32]. This holistic method of survey development emphasizes placing attention on all aspects of development, implementation, and follow-up to improve the experience of the respondent and increase likelihood of response [32]. Founded around theories of social exchange, this method outlines best practices to help survey designers reduce costs (financial, time, etc.), increase benefits (e.g., implications for respondent, compensation, etc.) and build credibility and trust with respondents.

In an attempt to control survey error for nonresponse and build trust with respondents, researchers chose to administer through the survey through Texas A&M AgriLife Extension Agents, under the guidance of Regional Program Leaders (RPL) in the Agriculture and Natural Resources (ANR) program units [32]. These RPLs oversee ANR Extension Agents located in the West and East regions of the state who have well-established relationships with producers in their area. Because of potential sensitivities around mental health and mental illness, administering the survey through this well-known state agency via personal emails from Extension Agents—who potential respondents deemed credible and trustworthy—served as an additional control or proactive attempt to control for nonresponse.

Once buy-in from RPLs and Extension Agents was obtained, implementation policies and procedures were explicitly explained and agreed upon by the RPLs overseeing distribution for their units and procedures were relayed to Extension Agents. Prior to survey administration, the two RPLs received requests over email and phone to help obtain the email listservs of ANR extension agents for both regions and prepare their agents for survey distribution. During this time, the RPLs contacted their Extension Agents, both in person and via email, to encourage cooperation, per Dillman et al.’s recommendation for mixed-modal data collection [32] to reduce survey error. Initial contact was then made with the Extension Agents in an email. In this email, researchers provided agents with information about the potential impact [32] of these survey results within their communities—to garner their support. Following initial contact, protocols were sent to both RPLs and Extension Agents with information regarding survey distribution. On each day of distribution, researchers sent Extension Agents the appropriate recruitment emails, which encouraged producers in their regions to participate. Included in the recruitment email was a rich description, depicting the impact of survey results within communities and a specific nod to the study’s sponsorship [32] by Texas A&M AgriLife Extension Service. Researchers and RPLs also sent same-day follow-ups, reminding Agents to forward recruitment emails to their listservs, if they had not yet done so. Per Dillman et al.’s tailored design method [32], potential participants received five contact points during survey implementation. Throughout this entire phase, routine communication between RPLs and researchers occurred to eliminate potential confusion and control for any deviation in distribution protocol.

In adherence to Dillman et al., who outlines the advantage of an incentive to increase response [32], study designers collaborated with a county Farm Bureau to obtain two $100 gift cards. Respondents were given the opportunity to provide contact information at the end of the survey to be entered into a drawing for one of the gift cards. Winners were contacted via phone or email and received their gift card in the mail.

### 2.4. Statistical Analyses

Researchers calculated frequencies and percentages for demographic data in order to describe producer characteristics. To better understand distribution of the data, means, grand means, and standard deviations for demographic information, social identity, social capital, and self-stigma and general help-seeking for personal or emotional problems and for suicide ideation were calculated. A Pearson’s r correlational coefficient was used to determine the relationship between social identity, social capital, and self-stigma. To test effects of the variables (i.e., social identity, social capital, and self-stigma) on help-seeking intention for personal or emotional problems and for suicide ideation, researchers conducted multiple linear regression analyses.

## 3. Results

### 3.1. Relationship between Social Identity, Social Capital, and Self-Stigma

Results revealed a negative, moderate association between social identity and social capital, (r = −0.33, N = 346, *p* < 0.001). However, it is important to note that lower means on the PSCS represented a larger social capital. Correlations for self-stigma were not significant with either social identity or social capital.

### 3.2. Variables Influencing Help-Seeking Intention for Personal or Emotional Problems

Researchers conducted a multiple linear regression to determine the effect of social identity, social capital, and self-stigma on help-seeking for personal or emotional problems. Results from the multiple linear regression indicated that statistically significant relationships existed between seeking help for personal or emotional problems and social capital and self-stigma The regression model using social identity, social capital, and self-stigma as predictors accounted for 24% of the variance in producers’ help-seeking intentions for personal or emotional problems, according to the R^2^ value. The model explained a statistically significant amount of variation in the outcome (*F*(3, 308) = 31.80, *p* = 0.01). Help-seeking for personal or emotional problems was predicted by social capital (*t*(308) = −7.10, *p* = 0.01) and self-stigma (*t*(308) = −5.76, *p* = 0.01). It is important to note scores for social capital were reverse coded and thus, lower scores within the construct actually represented a higher social capital. On average, one standard deviation increase in social capital is associated with 0.38 standard deviation increase in help-seeking intentions for personal or emotional problems, and one standard deviation increase in self-stigma is associated with 0.29 standard deviation decrease in help-seeking intentions for personal or emotional problems. Coefficients for social identity were not statistically significant (*p* = 0.58). The data from this statistical test are displayed in Table 1.

### 3.3. Variables Influencing Help-Seeking Intention for Suicide Ideation

Researchers administered a second multiple linear regression to test the effect of social identity, social capital, and self-stigma on help-seeking for suicidal thoughts and confirmed statistically significant relationships. These results were similar to those presented above. The regression model using social identity, social capital, and self-stigma as predictors accounted for 15% of the variance in producers’ help-seeking intentions for suicide ideation, according to the R^2^ value. The model explained a statistically significant amount of variation in the outcome (*F*(3, 291) = 16.42, *p* = 0.01). Help-seeking for suicide ideation was predicted by social capital (*t*(291) = −6.24, *p* = 0.01) and self-stigma (*t*(291) = −2.71, *p* = 0.01). Again, lower scores on the construct for social capital represented higher levels of social capital. On average, one standard deviation increase in social capital is associated with 0.36 standard deviation increase in help-seeking intentions for suicide ideation and one standard deviation increase in self-stigma is associated with 0.15 standard deviation decrease in help-seeking intentions for suicide ideation. Coefficients for social identity were not statistically significant (*p* = 0.55). Results from this statistical test are displayed in Table 2.

## 4. Discussion

This study first sought to investigate the relationship between social identity, social capital, and self-stigma. Researchers were able to confirm a relationship between social identity and social capital, which aligns with previous literature [14]. While an initial look at the correlation might suggest that social identity and social capital were negatively correlated, since scores for social capital were reverse coded, lower scores actually represented a higher social capital. This suggests that the greater emphasis respondents’ placed on social identity, the higher their scores for social capital. This aligns with previous research which suggests that a greater sense of social identity can expand producers’ social networks [14]. When producers identify broadly to their social group—and that identity is salient—this social group can positively impact their help-seeking behavior, when others in their social networks promote progressive health behavior [10,34]. These findings support communication efforts in the industry to help shift rural, agrarian paradigms to encourage a culture of health-promoting behaviors, such as accepting support, and suggest need for cultivating intentional community among producers, starting with those willing to be advocates for mental health. Targeted efforts, through formal or informalized educational programs and advertising campaigns, to reverse health-debilitating norms and stigmas reinforced by individuals within the agriculture industry, would also be a valuable investment of time and resources. These would be especially impactful if led by agricultural leaders, agribusinesses and stakeholder organizations of influence, such as federal and state agencies and national organizations or commodity groups.

Second, this study examined the effect of social identity, social capital, and self-stigma on agricultural producers’ intention to seek assistance in the event of personal or emotional problems and suicide ideation. The regression models were determined to be a good fit, using the R^2^ measure. The R^2^ delineates the percent variance in a dependent variable explained by all independent variables in our study. Researchers concluded that social identity is not a significant predictor of help-seeking intention. Results indicated that greater levels of social capital were associated with higher intentions to seek help for personal or emotional problems and for suicide ideation. Further, findings on the positive influence of social capital supported previous studies, which found that individuals engage in healthy help-seeking behavior when they receive positive encouragement from individuals in a social network [8,14,15]. This is especially true of rural individuals with high levels of bonding social capital, where strong, close relationships exist among most, if not all members of a social group or community, such as family or close friends [16,17]. Specific attention should be paid to those within inner circles of support, such as spouses and significant others and immediate family members, whose proximity and investment in their loved one’s wellbeing might disproportionately affect their own mental health [8]. Thus, we recommend that awareness campaigns and crisis prevention training be developed to support, equip, and empower these family members who might find themselves on the frontline of intervention. Additionally, solutions aiming to increase social capital, widen social networks, and provide various outlets of support outside of family and close friends would be valuable and effective for increasing producers’ intention to seek help, promoting positive health behaviors. This could be achieved through local or regional support groups, community events and policy aimed at enhancing support groups and educational resources for family members and community leaders in rural areas.

In contrast, this research found that agricultural producers with higher self-stigma tend to have lower intention to seek mental health assistance. These findings were consistent with previous literature, which stated that self-stigma largely contributes to help-seeking resistance regarding mental health issues [18,28]. One way to combat and destigmatize mental health is by increasing mental health literacy [35]. Rural health practitioners should consider what partnerships and collaborations could be developed to offer education and mental health literacy programming for individuals in the agricultural industry, especially agricultural producers, and those within their proximal social network. Policy could be created to ensure that mental health programming and resources are made accessible in rural areas, allowing rural practitioners, community leaders and appropriate agricultural industry representatives to complete Mental Health First Aid (MHFA), or a related course. Evidence suggests MHFA and related trainings contribute to the destigmatization of mental health in rural areas and contribute to expanding infrastructure for rural mental healthcare [36] and behavioral health services. This includes improving access to pop-up clinics, telehealth, and traveling counselors in rural areas where standard mental healthcare might not otherwise be available.

Further, agricultural leaders, rural agribusinesses, and organizations should utilize their platforms to talk openly and honestly about farm stress and mental illness in rural populations. Agricultural extension providers are structured for quick responses to farmers needs and demands [37,38]. Opportunity exists to address and train youth to be propellants that help shift industry views. Rural youth organizations such as National FFA Association and National 4-H can capitalize on their youth, adolescent, and young adult audiences and equip them with the knowledge, resources, and skills they need to better prioritize their mental health and support their peers. It is recommended that these organizations look to how they can develop and implement mental health curriculum into their programming to raise mental health awareness and literacy amongst their members to eradicate stigma as a barrier.

### Limitations

This study utilized self-reported data, which can introduce potential error and could lead to measurement bias, if not controlled [39]. In addition, the accessible sample was restricted to agriculturalists in the East and West ANR Regions in Texas. While the sampled population did closely mirror the demographic makeup of Texas producers, future researchers should consider repeating this study statewide to obtain a more representative sample. Consideration should also be given to repeat the study in other states, or even nationwide, to allow for increased generalizability to the broader population of agricultural producers. Additionally, to access the population and increase survey response according to Dillman et al. [32] the survey was administered electronically. While not an evident limitation, given that the majority of respondents were over 65 years of age, basic working knowledge, access or technology intimidation/anxiety [40] might have made it more difficult or inhibited them from completing the survey. To control for potential nonresponse error, a strict and regimented survey distribution protocol was developed to increase likelihood of participation. Survey instructions were detailed and checked for clarity to mitigate confusion. Additionally, participants were given the opportunity to contact researchers if they had questions or needed assistance accessing the survey. However, if desired, alternative survey forms could be considered.

## 5. Conclusions

This study provides empirical evidence that social capital and self-stigma are significant predictors of agricultural producers’ intention to seek help for personal or emotional problems and for suicide ideation. Based on these findings, advocacy groups and local policy makers should consider ways to provide support and resources to address and lower self-stigma in agricultural producers and aid in increasing their social capital. Additionally, if they are not currently, rural health practitioners should consider working with agricultural producers and local industry-based organizations or institutions (e.g., Farm Bureau federations, Extension agencies, colleges of agriculture and related disciplines at land-grant universities) to better understand their unique challenges and needs. These efforts could ultimately increase intention and instigate positive help-seeking behavior. If improved help-seeking is achieved, these efforts have potential to help agricultural producers achieve improved mental health outcomes. This research contributes modern, relevant findings to a previously dated and narrow body of literature on mental health help-seeking among rural agriculturalists and underscores the importance for continued research surrounding mental health and help-seeking among agricultural producers, a historically understudied and underserved population regarding mental health.

## Figures and Tables

**Table 1:** Analysis of multiple regression results of independent variables on help-seeking for personal or emotional problems.

	Unstandardized Coefficients		Standardized Coefficients		
Model	*B*	Std. Error	Beta	*t*	Sig. ^1^
(Constant)	6.29	0.49	22.02	12.68	0.01 *
Social Identity	0.04	0.07	0.03	0.55	0.58
Social Capital	−0.65	0.09	−0.38	−7.10	0.01 *
Self-Stigma	−0.36	0.06	−0.29	−5.76	0.01 *

^1^ * *p* < 0.01.

**Table 2:** Analysis of multiple regression results of independent variables on help-seeking for suicidal thoughts.

	Unstandardized Coefficients		Standardized Coefficients		
Model	*B*	Std. Error	Beta	*t*	Sig. ^1^
(Constant)	6.60	0.70		9.43	0.01 *
Social Identity	−0.06	0.09	−0.04	−0.61	0.55
Social Capital	−0.80	0.13	−0.36	−6.24	0.01 *
Self-Stigma	−0.24	0.09	−0.15	−2.71	0.01 *

^1^ * *p* < 0.01.

## Data Availability

The datasets used and/or analyzed during the current study are available from the study group on reasonable request. Please contact the corresponding author.

## Abbreviations

ANR	Agricultural and Natural Resources
CDC	Center for Disease Control
CFR	Code of Federal Regulations
COIC	Collective Occupational Identity Construct
GHSQ	General Help Seeking Questionnaire
PSCS	Personal Social Capital Scale
RPL	Regional Program Leader
SSOSH	Self Stigma of Seeking Help
